# Innovations in the Realm of Shoulder Arthroplasty

**DOI:** 10.3390/jcm12010237

**Published:** 2022-12-28

**Authors:** Alexandre Lädermann, Laurent Audigé, Markus Scheibel

**Affiliations:** 1 Division of Orthopaedics and Trauma Surgery, La Tour Hospital, Av. J.-D. Maillard 3, 1217 Meyrin, Switzerland; 2 Faculty of Medicine, University of Geneva, 1211 Geneva, Switzerland; 3 Division of Orthopaedics and Trauma Surgery, Department of Surgery, Geneva University Hospitals, 1211 Geneva, Switzerland; 4 Department of Shoulder and Elbow Surgery, Schulthess Clinic, 8008 Zurich, Switzerland; 5 Research and Development Department, Schulthess Clinic, 8008 Zurich, Switzerland; 6 Department of Orthopaedic Surgery and Traumatology, University Hospital of Basel, 4031 Basel, Switzerland; 7 Center for Musculoskeletal Surgery, Campus Virchow, Charité-Universitaetsmedizin Berlin, 10117 Berlin, Germany

## Introduction

Most of the surgeries regarding the shoulder were established over a century ago. In the 1890s, the understanding of the unstable shoulder was elucidated by Broca and Hartman [1], who introduced the concept of capsulolabral damage following dislocations as a possible cause of recurrent instability [2]. Notably, most of the findings currently considered hallmarks of shoulder instability, including Bankart lesions, bony Bankart lesions, and Kim lesions, as well as anterior and posterior labral periosteal sleeve avulsions and glenoid avulsions of glenohumeral ligaments, were described within research papers decades before their depiction by the eponymous figures to whom these lesions are now commonly assigned [2]. In 1906, Perthes [3] and, a few years later, Bankart [4], emphasized the reattachment of the labrum to stabilize the joint. Current bone grafting techniques are based on the initial descriptions by Noeske in 1921 using the coracoid process [5], Eden [6] in 1918, and Hybinette [7] in 1932, using an autologous iliac crest. Since then, no true paradigm shift has occurred.

Regarding the rotator cuff, a similar observation can be made. Duplay presented the classic description of scapulohumeral periarthritis in 1872, highlighting the potential role of the acromion. Repair of the torn rotator cuff likely dates back to 1898 [8]. Since then, many evolutions regarding these treatments, such as acromioplasty, arthroscopy, or anchors development, have been subsequently observed, but without apparent revolution; 150 years after its first description, the proper place of a procedure such as acromioplasty has yet to be determined [9], and most enhancing technologies (superior capsular reconstruction (SCR) [10], growth factors (PRP) [11], Balloon [12], etc.) for rotator cuff reinforcement or substitution have yet to prove their superiority over simple reattachment of the tendon to the bone.

Interestingly, the former statements are not true within the domain of arthroplasty. Since Themistocles Gluck designed the first shoulder prostheses in 1890, of which Jules Emile Péan implanted the first in 1893 [13], several revolutions have taken place within these last few decades, namely, by Charles Neer and Paul Grammont. Most importantly, the realm of shoulder arthroplasty has undergone significant transformation [14] in recent years, covered in the present Special Issue on shoulder arthroplasty in the *Journal of Clinical Medicine*. It concerns not only surgical indications that have dramatically evolved [15,16,17], but also planification and navigation with the implementation of artificial intelligence (AI) and augmented reality (AR) [18]. Moreover, the rapid development of surgical techniques [19,20] and new prosthetic designs [21,22,23], including custom augments with three dimensional (3D) printing, glenoid [24] and humeral [25] reconstruction for various conditions [26], are overviewed. Palpable results of this recent technologic acceleration include improved outcomes [27] and decreased complication rates. Despite the significant progress highlighted in this Special Issue, there is currently a myriad prosthetic designs announcing imminent changes. Indeed, we are only at the dawn of a new era in the history of shoulder arthroplasty, reminding us that a substantial amount of work remains to be carried out in order to see progress. 
